# Rapid experimental SAD phasing and hot-spot identification with halogenated fragments

**DOI:** 10.1107/S2052252515021259

**Published:** 2016-01-01

**Authors:** Joseph D. Bauman, Jerry Joe E. K. Harrison, Eddy Arnold

**Affiliations:** a Center for Advanced Biotechnology and Medicine, Department of Chemistry and Chemical Biology, Rutgers University, 679 Hoes Lane, Piscataway, NJ 08854, USA

**Keywords:** phasing, drug discovery, X-ray crystallography

## Abstract

Through X-ray crystallographic fragment screening, 4-bromopyrazole was discovered to be a ‘magic bullet’ that is capable of binding at many of the ligand ‘hot spots’ found in HIV-1 reverse transcriptase (RT). The binding locations can be in pockets that are ‘hidden’ in the unliganded crystal form, allowing rapid identification of these sites for *in silico* screening. In addition to hot-spot identification, this ubiquitous yet specific binding provides an avenue for X-ray crystallographic phase determination, which can be a significant bottleneck in the determination of the structures of novel proteins. The anomalous signal from 4-bromopyrazole or 4-iodopyrazole was sufficient to determine the structures of three proteins (HIV-1 RT, influenza A endonuclease and proteinase K) by single-wavelength anomalous dispersion (SAD) from single crystals. Both compounds are inexpensive, readily available, safe and very soluble in DMSO or water, allowing efficient soaking into crystals.

## Abstract 2

4-Bromopyrazole and 4-iodopyrazole bind to many small molecule binding hot spots in target proteins. This promiscuous binding enables the use of these compounds for experimental phase determination by single-wavelength anomalous dispersion (SAD). The low cost and safety of the compounds make them excellent choices for addition to the protein crystallographer’s toolkit.

## 1. Introduction   

Solving three-dimensional protein crystal structures without knowledge of related structures or with minimal model bias requires experimental determination of phases, which is often accomplished using single-wavelength anomalous dispersion (SAD) or multi-wavelength anomalous dispersion (MAD) (Taylor, 2010 ▸). The popularity of these techniques has been due, in large part, to improvements at synchrotron and home X-ray sources that allow the collection of very high-quality data from single crystals. Since the anomalous signals from native atoms are relatively weak, it is normally advantageous to add heavier atoms to protein crystals either through incorporation during protein expression (*i.e.* selenomethionine; Hendrickson *et al.*, 1990 ▸), co-crystallization as a binding partner (Chen *et al.*, 1991 ▸) or soaking into previously formed crystals (Dauter *et al.*, 2000 ▸; Beck *et al.*, 2010 ▸; Abendroth *et al.*, 2011 ▸). Of the three, soaking atoms or compounds into preformed crystals is the least laborious, but multiple attempts using different heavy-atom derivatives may be necessary to find a derivative with sufficient binding occupancy in the target crystal. Compounds containing multiple heavy atoms, including 5-amino-2,4,6-triiodoisophthalic acid (Beck *et al.*, 2008 ▸), the 12-tungstophosphate cluster (Rudenko *et al.*, 2003 ▸) and Ta_6_Br_12_ (tantalum cluster; Knäblein *et al.*, 1997 ▸), have been developed in order to maximize anomalous signal and are capable of binding to a wide variety of proteins. These compounds were optimized using the maximum number of anomalous signal-producing atoms at the cost of compound volume/complexity and therefore potential binding sites.

Hits from small-molecule screening tend to bind to one or multiple hot spots within a target protein (DeLano, 2002 ▸; Barelier *et al.*, 2010 ▸). Several computational approaches have been developed in order to find these hot spots for further *in silico* screening (Kozakov *et al.*, 2015 ▸; Morrow & Zhang, 2012 ▸). However, these cannot adequately account for protein dynamics in predicting transient protein/ligand pockets in apo crystals.

In fragment-based drug discovery, ‘fragments’ are defined simply as less complex drug-like molecules that have a mass of less than 250 Da and are associated with higher hit rates relative to the larger drug-like molecules typically used in high-throughput screening. Multiple experimental approaches for hot-spot identification have also been developed using either high concentrations of various solvents (Mattos & Ringe, 1996 ▸) or small libraries of fragments (18–150 compounds; Barelier *et al.*, 2010 ▸; Grøftehauge *et al.*, 2013 ▸). The small-fragment library approach has been further enabled by the observation of high hit rates from halogenated fragments for several target proteins (Grøftehauge *et al.*, 2013 ▸; Bauman, Patel, Dharia *et al.*, 2013 ▸; Tiefenbrunn *et al.*, 2014 ▸). Experimental approaches have the added benefit of being able to identify ‘hidden pockets’ that are not readily observable in the unliganded form of the protein and yet whose population can be increased by the binding of a compound to the transient pocket. However, experimental approaches require many data sets to be collected following crystal soaks, as well as soaking optimization and/or co-crystallization experiments to map the small-molecule binding sites for a target protein.

**Figure 1:**
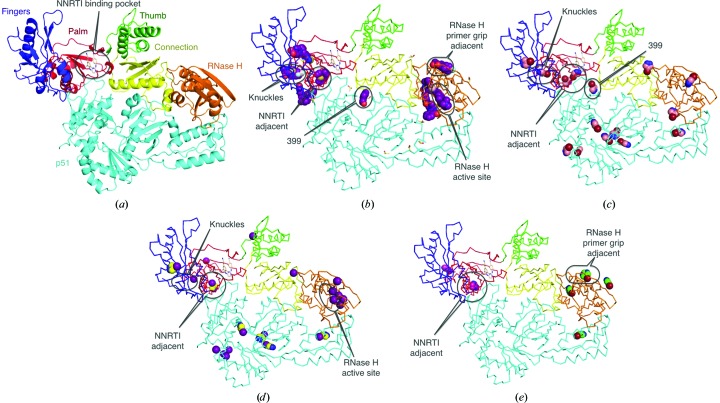
Hot-spot identification for HIV-1 RT–rilpivirine (figures drawn using *PyMOL*; DeLano, 2006 ▸). (*a*) The RT–rilpivirine structure with p66 subdomains colored and labeled and the p51 subunit colored cyan. Rilpivirine is depicted at the NNRTI-binding pocket. (*b*) Results of an X-ray crystallographic fragment-screening campaign on RT–rilpivirine crystals not including 4-bromopyrazole (Bauman, Patel, Dharia *et al.*, 2013 ▸). Compounds located are depicted as purple spheres and the sites relevant to this study are labeled. (*c*) Locations of 4-bromopyrazole are shown as light pink spheres. The locations corresponding to compound-binding hot spots identified in the screening of 775 compounds are labeled. (*d*) Locations of 4-iodopyrazole are shown as yellow spheres. The positions where the I atoms are present based on anomalous signal are shown as purple spheres. (*e*) The sites where iodopyrazine (pink spheres) and 5-bromopyrimidine (green spheres) bound are depicted with labels for the previously identified hot spots.

During an X-ray crystallographic small-molecule fragment screening campaign with 775 compounds targeting HIV-1 reverse transcriptase (HIV-1 RT; Fig. 1 ▸ *a*) bound to a non-nucleoside reverse transcriptase (NNRTI) drug, rilpivirine (RT–rilpivirine; Fig. 1 ▸ *b*; Bauman *et al.*, 2012 ▸; Bauman, Patel, Dharia *et al.*, 2013 ▸), 4-bromopyrazole was found to bind to 15 sites (Fig. 1 ▸ *c*). These sites were found throughout two subunits of HIV-1 RT: p66 (comprising fingers, palm, thumb, connection and RNase H subdomains) and p51 (Fig. 1 ▸ *c*). 4-Bromo­pyrazole was found at several ligand-binding hot spots discovered through separate fragment-screening experiments, including the newly identified allosteric pockets referred to as the Knuckles (at the fingers/palm junction), NNRTI Adjacent (near the NNRTI-binding pocket in the palm subdomain) and RNase H Primer Grip Adjacent (near the RNase H active site) sites.

A single compound partially reproducing the comprehensive binding-site array identified from a 775-compound library led us to consider 4-bromopyrazole for rapid hot-spot identification. While exploring this hypothesis, we also recognized the potential of using 4-bromopyrazole and 4-iodopyrazole for SAD phasing. Here, we report the use of 4-bromopyrazole and 4-iodopyrazole in hot-spot identification and to solve the structures of HIV-1 RT, influenza A endonuclease and proteinase K by SAD phasing from single crystals.

## 2. Methods   

### 2.1. Crystallization   

HIV-1 RT was expressed and purified as described previously (Bauman, Patel, Dharia *et al.*, 2013 ▸; Supporting Information). Crystallization of RT was performed using the sitting-drop method in 96-well crystallization trays (Qiagen, Valencia, California, USA). Prior to crystallization, the HIV-1 RT construct RT52A (20 mg ml^−1^; Bauman, Patel, Dharia *et al.*, 2013 ▸) was incubated with rilpivirine (TMC278/Edurant) at a 1:1.5 protein:drug molar ratio at room temperature (∼23°C) for 30 min. RT–rilpivirine crystals were produced in hanging drops at 4°C with a 1:1 ratio of protein solution and well solution consisting of 11%(*v*/*v*) PEG 8000, 4%(*v*/*v*) PEG 400, 50 m*M* imidazole pH 6.6, 10 m*M* spermine, 15 m*M* MgSO_4_, 100 m*M* ammonium sulfate, 5 m*M* tris(2-carboxyethyl)phos­phine together with an experimentally optimized concentration of microseeds from previously generated and crushed RT–rilpivirine crystals (pre-seeding).

Influenza endonuclease was expressed, purified and crystallized as described previously (Bauman, Patel, Baker *et al.*, 2013 ▸). The endonuclease was crystallized using the sitting-drop method in 96-well crystallization trays (Qiagen, Valencia, California, USA). 5 mg ml^−1^ endonuclease was mixed with an equal volume of precipitant solution consisting of 1 m*M* manganese chloride, 200 m*M* MES pH 6.7, 25%(*v*/*v*) PEG 8000, 100 m*M* ammonium sulfate, 10 m*M* magnesium acetate, 10 m*M* taurine, 50 m*M* sodium fluoride. All crystallization was performed at 20°C.

Proteinase K was purchased from Sigma–Aldrich (St Louis, Missouri, USA) and crystallized as described previously (Beck *et al.*, 2010 ▸). 20 mg ml^−1^ proteinase K was mixed with an equal volume of 100 m*M* Tris pH 7.2, 1.28 *M* ammonium sulfate.

### 2.2. Ligand soaks   

Compounds were purchased from Sigma–Aldrich (St Louis, Missouri, USA) or Acros (Geel, Belgium).

The RT/compound/cryosoaking solutions were prepared using crystallization well solution with the addition of 80 m*M* l-arginine, 5%(*v*/*v*) ethylene glycol and 20%(*v*/*v*) DMSO (containing the compound at the indicated concentrations between 20 and 500 m*M*). 80 m*M* l-arginine was included to improve the solubility of the ligands (Bauman, Patel, Dharia *et al.*, 2013 ▸). Crystals of RT52A–rilpivirine were harvested three months after they formed. The crystals were placed in compound/cryosoaking drops for 10 min before flash-cooling in liquid nitrogen.

Influenza endonuclease ligand soaks were performed by gradient shifting, over 20 min, the crystal solution to 1 m*M* manganese sulfate, 200 m*M* HEPES pH 7.7, 25%(*w*/*v*) PEG 8000, 50 m*M* ammonium sulfate, 5 m*M* magnesium acetate, 10%(*v*/*v*) DMSO, 5%(*v*/*v*) ethylene glycol. Compounds in DMSO were soaked into crystals (final volume of 10%) for 2 h before flash-cooling in liquid nitrogen.

Proteinase K ligand soaks were performed by the addition of 500 m*M* 4-iodopyrazole directly to the well solution with the addition of 30%(*v*/*v*) glycerol for cryoprotection. Crystals were soaked in the ligand solution containing cryoprotectant for 10 min before flash-cooling in liquid nitrogen.

### 2.3. Data collection and processing   

X-ray diffraction data collection was performed on the F1 beamline at the Cornell High Energy Synchrotron Source (CHESS), beamline X25 at the National Synchrotron Light Source (NSLS) and the CABM Macromolecular X-ray Crystallography Facility. The diffraction data were indexed, processed, scaled and merged using *HKL*-2000 (Otwinowski & Minor, 1997 ▸). Experimental phasing, structure refinement and model building were carried out using *PHENIX* (v.1.9-1692; Adams *et al.*, 2010 ▸) and *Coot* (v.0.8.1; Emsley *et al.*, 2010 ▸). Data-collection and processsing statistics are given in Table 1 ▸.

### 2.4. DFT calculations   

The geometry was fully optimized for each compound in its singlet ground state using the M06 functional (Zhao & Truhlar, 2008 ▸) as implemented in *Spartan*’14 v.1.1.0 (Wavefunction Inc., Irvine, California, USA) with the 6-311+G** basis set, which in the valence space is of triple-zeta quality and of double-zeta quality polarization functions (Glukhovtsev *et al.*, 1995 ▸; Yao *et al.*, 2007 ▸; Ribeiro *et al.*, 2012 ▸; Shallangwa *et al.*, 2014 ▸). The structures were verified to be at a minimum energy without any imaginary frequencies. Single-point energy calculations were computed using the 6-311++G** basis set and the optimized structures with a diffuse function on all of the atoms, including the H atoms. Electrostatic potential energy maps were generated at a 0.002 (arbitrary units) isosurface. The electrostatic surface potential (ESP) represents a charge-density distribution which gives a visual indication of probable interactions of a point-like charged species with organic molecules (Naray-Szabo & Ferenczy, 1995 ▸; Mircescu *et al.*, 2011 ▸).

## 3. Results and discussion   

### 3.1. 2-Bromopyrazole derivatives   

To determine whether the observed promiscuous binding was solely limited to 4-bromopyrazole, analogs were purchased and screened at concentrations of 20 m*M* (the soaking concentration for 4-bromopyrazole) and 500 m*M* (the full list is given in Supplementary Fig. S1). Of the 20 halo­genated single-ring compounds screened at 20 m*M*, only 4-iodopyrazole bound to RT–rilpivirine in a manner comparable to 4-bromopyrazole (Fig. 1 ▸ *d*). 4-Iodopyrazole bound to 21 sites in total, but only five of these sites showed sufficient electron density for reliable modeling of the full compound. The 11 remaining sites were identified based on anomalous signal and modeled as single I atoms (Fig. 1 ▸ *d*). This result is particularly promising as 4-iodopyrazole allows SAD experiments to be performed conveniently at the Cu *K*α edge (λ = 1.5418 Å) normally available on home X-ray sources, as Δ*f*′′ for iodine is ∼7 electrons at this energy. Two additional compounds, 2-bromopyrimidine and iodopyrazine, bound to four and two sites, respectively, at a higher soaking concentration of 500 m*M*. 2-Bromopyrimidine was found at the RNase H Primer Grip Adjacent site, while iodopyrazine was located in the NNRTI Adjacent site in the same position as both 4-iodopyrazole and 4-bromopyrazole (Fig. 1 ▸ *e*).

**Figure 2:**
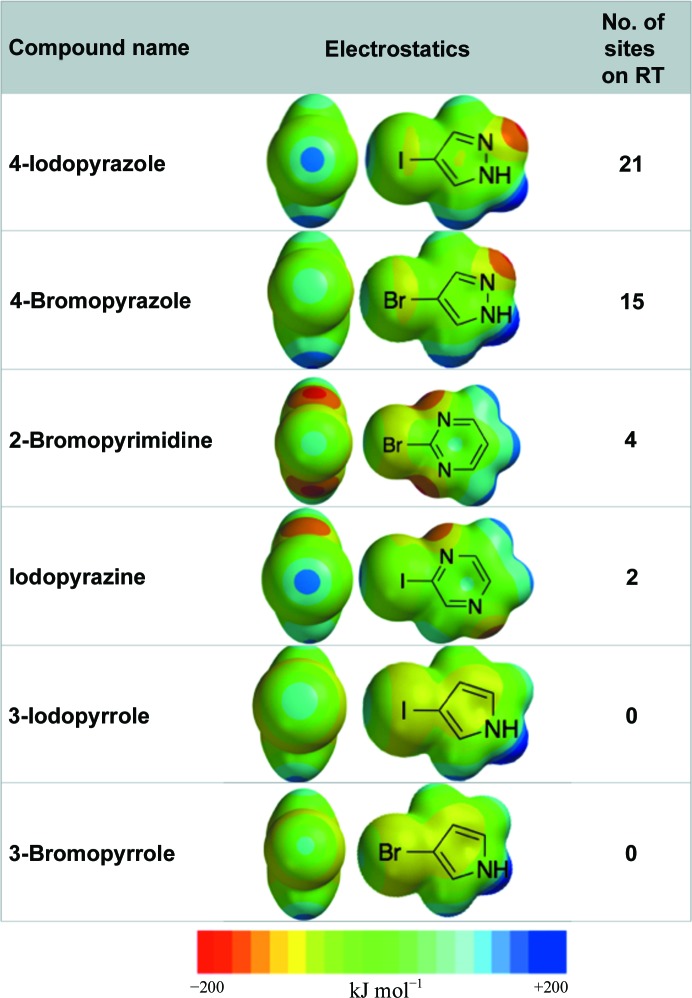
Computed electrostatic surfaces for 4-bromopyrazole, 4-iodopyrazole and related analogs. Partial table of derivatives tested for binding to RT–rilpivirine with density functional theory calculations of the electrostatic potential energy surface shown (a full list of derivatives can be found in the Supporting Information).

We hypothesized that the promiscuous nature of 4-bromopyrazole and 4-iodopyrazole may be owing to the electrostatics and small size of these compounds. These compounds are capable of creating diverse interactions with protein and solvent atoms through a basic chemical scaffold consisting of six non-H atoms that form an aromatic ring with hydrogen-bond donor and acceptor functionality and a halogen, providing diverse contacts with protein atoms and solvent molecules. Density functional theory calculations revealed that the electronegative N atoms in the pyrazole ring enhance the polarization of the Br atom in both negative charge and the positive charge of the ‘sigma hole’ opposite to the C—Br bond when compared with less electronegative heterocyclic rings (Fig. 2 ▸ and expanded list in Supplementary Fig. S1; Auffinger *et al.*, 2004 ▸; Wilcken *et al.*, 2013 ▸).

### 3.2. Hot-spot identification   

**Figure 3:**
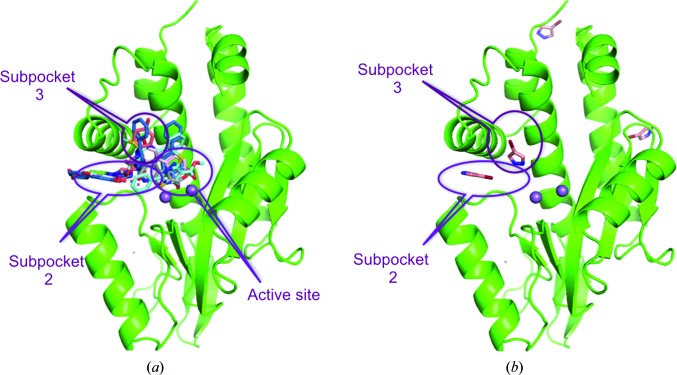
Hot-spot identification for influenza endonuclease. (*a*) Results of a crystallographic fragment-screening campaign on influenza endonuclease. Small molecules found are depicted as sticks and the locations that were named are labeled. The active-site metal ions (Mn^2+^) are shown as purple spheres. (*b*) Sites where 4-­bromopyrazole was located in endonuclease with labels for subpockets 2 and 3.

The potential use of 4-bromopyrazole or 4-iodopyrazole for the identification of binding hot spots was further explored by soaking the compounds at 500 m*M* into protein crystals of RT–rilpivirine, pandemic 2009 influenza N-terminal PA endonuclease (Bauman, Patel, Baker *et al.*, 2013 ▸) and proteinase K from *Tritirachium album* Limber (Betzel *et al.*, 1988 ▸; Beck *et al.*, 2010 ▸). Fragment screening by X-ray crystallography has previously been performed on influenza endonuclease (Bauman, Patel, Baker *et al.*, 2013 ▸), allowing a comparison of the hot-spot identification from a 775-compound library and the binding-site identification using 4-bromopyrazole (Fig. 3 ▸ *a*). 4-Bromopyrazole bound clearly to four locations within the endonuclease, including two of the three sites (subpockets 2 and 3) that were identified as hot spots by previous fragment-screening experiments (Fig. 3 ▸ *b*).

**Figure 4:**
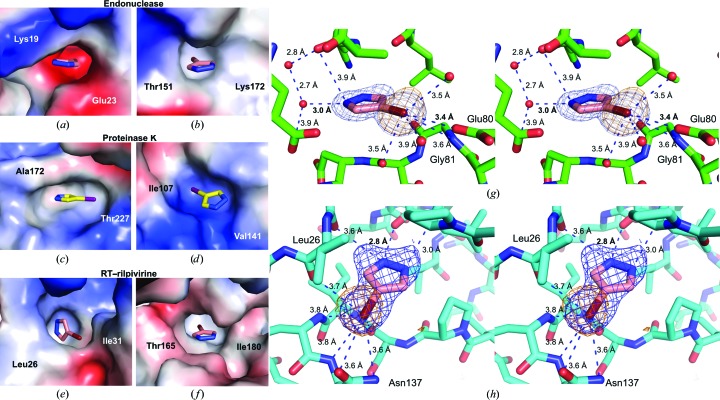
4-Bromopyrazole and 4-iodopyrazole binding sites. (*a*)–(*f*) Two binding sites with the clearest electron density for the compounds are depicted for each protein: (*a*, *b*) influenza endonuclease, (*c*, *d*) proteinase K and (*e*, *f*) RT–rilpivirine. Electrostatic potential surfaces are shown. Two residues in each pocket are labeled. (*g*, *h*) Wall-eye stereoviews of 4-bromopyrazole bound to pockets in influenza endonuclease (*g*) or RT–rilpivirine (*h*). Potential electrostatic interactions are depicted as blue dashes with interatomic distances shown. Compound OMIT maps are contoured at 3.0σ (blue; *F*_o_ − *F*_c_ coefficients) and anomalous difference density is contoured at 5.0σ [orange mesh; *F*(*hkl*) − *F*(−*h*−*k*−*l*) coefficients].

From each protein soak, the two sites with the clearest electron density (based on electron-density coverage of the compound) for the compound and surrounding residues were analyzed for types of interactions. Remarkably, the types of sites with compound binding spanned from mildly electropositive through hydrophobic to very electronegative as the amphiphilic halogen can form hydrophobic and polar (both positive or negative) interactions owing to its high polarizability (Figs. 4 ▸ *a*–4 ▸ *h*; Auffinger *et al.*, 2004 ▸; Metrangolo *et al.*, 2007 ▸; Parisini *et al.*, 2011 ▸; Sirimulla *et al.*, 2013 ▸). Adding to the binding repertoire of the compound, the pyrazole N atoms also formed electrostatic interactions with protein atoms either directly or through bridging waters (Figs. 4 ▸ *g* and 4 ▸ *h*).

### 3.3. Observation of ‘hidden’ ligand-binding sites   

**Figure 5:**
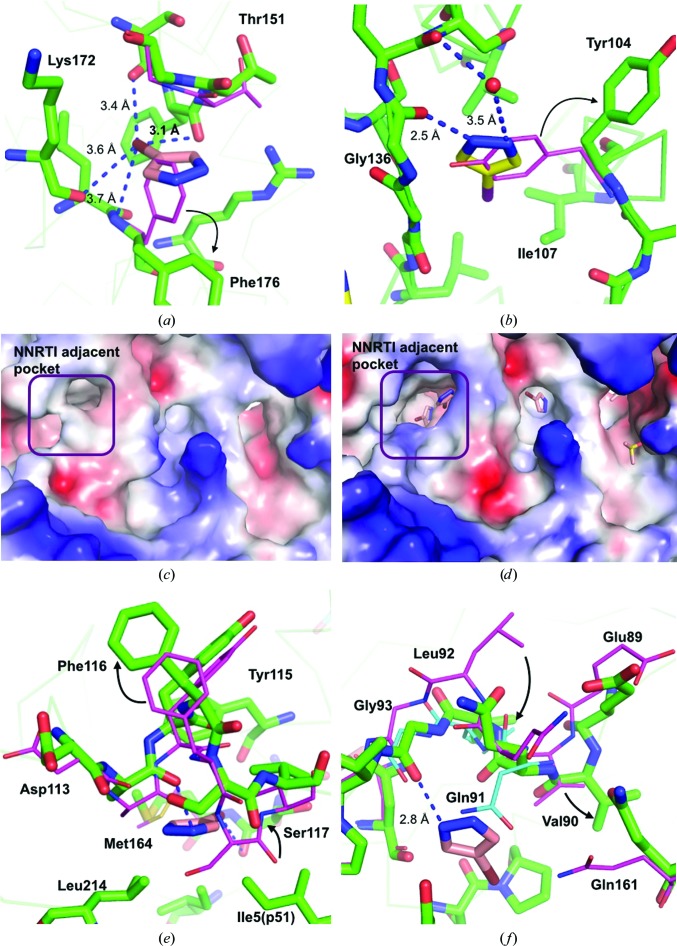
Hidden-pocket identification. Hidden pockets are shown for (*a*) influenza endonuclease, (*b*) proteinase K, (*c*, *d*) the HIV-1 RT nucleic acid-binding cleft, (*e*) the RT Knuckles site and (*f*) the RT NNRTI Adjacent site. (*a*, *b*) The positions of the apo residues are colored magenta and the bound form green. Major changes in positions are indicated with black arrows. (*c*, *d*) Electrostatic potential surface for the nucleic acid-binding cleft in RT–rilpivirine without (*c*) and with (*d*) 4-bromopyrazole. The location of the NNRTI Adjacent pocket is indicated. (*e*, *f*) The positions of the apo residues are colored magenta, the bound form green and the nucleic acid-bound form [in (*f*) only] cyan.

X-ray crystal structures provide a time-averaged snapshot of a protein structure, which exists in many conformations, including those that exist for a tiny fraction of the time and that are effectively invisible in the absence of ligands. Ligands that stabilize these fleeting conformations not only allow novel pocket identification by X-ray crystallography but may also inform and enable drug discovery. In the case of influenza endonuclease, binding of 4-bromopyrazole stabilized an alternate conformation of Phe176 with χ_1_ rotated 109° from its configuration in the apo structure (Fig. 5 ▸ *a*), opening an otherwise hidden hydrophobic site within the apo structure. There is also a 0.9 Å C^α^ backbone movement of Thr151 and Gly152, further expanding the pocket and allowing the binding of a small ligand.

In proteinase K, 4-iodopyrazole binds near the S4 protease recognition site (Fig. 5 ▸ *b*). The side chain of Tyr104 rotates χ_1_ by 180°, thereby creating a hydrophobic pocket in which the compound binds. Superposition of proteinase K structures deposited in the PDB indicates that this conformation of Tyr104 has not been reported previously, even though several compounds have been found that interact with Tyr104 in its apo conformation using X-ray crystallography (Wolf *et al.*, 1991 ▸; Betzel *et al.*, 1993 ▸). Since this conformation is clearly accessible, it provides new understanding and potential for new probe design for the substrate-recognition site of this well studied protein.

As we reported for RT–rilpivirine (Bauman, Patel, Dharia *et al.*, 2013 ▸), fragments are able to select and stabilize conformations that have not previously been detected in RT with rilpivirine or other inhibitors/substrates. Similar to what was previously shown with another fragment, the Knuckles pocket (Fig. 5 ▸ *e*) is stabilized in an open conformation formed by 2.7 Å C^α^ backbone shifts at Tyr115 and Phe116, which are key amino-acid residues involved in binding to the incoming dNTP during polymerization. A 20 m*M* concentration soak of 4-bromopyrazole also caused substantial residue movements along the nucleic acid-binding cleft of HIV-1 RT (Figs. 5 ▸ *c* and 5 ▸ *d*). The most dramatic changes occurred near the NNRTI Adjacent pocket, forming a larger pocket than that previously reported. Two molecules of 4-bromopyrazole bind in the extended NNRTI Adjacent site, with two additional molecules in a nearby site with p66 residues 89–93 separating them (Fig. 5 ▸ *f*). Binding of 4-bromopyrazole stabilized Leu92 in an alternative position with a C^α^ shift of 4.0 Å and a side-chain movement of 8.3 Å. Interestingly, the Leu92 position is similar to its location when RT is bound to a nucleic acid; however, the positions of the surrounding residues differ from those in published structures (Fig. 5 ▸ *f*; Das *et al.*, 2012 ▸).

### 3.4. SAD phasing with 4-bromopyrazole and 4-iodopyrazole   

**Figure 6:**
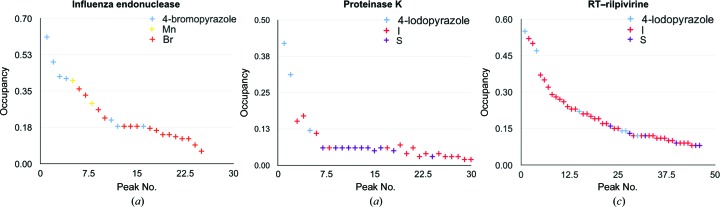
(*a*)–(*c*) Relative occupancies of anomalous sites determined by *AutoSol* colored by type of atom as assigned in the refined structure. (*a*) Sites found for influenza endonuclease with 4-bromopyrazole. The sites with highest occupancies show clear density for 4-bromopyrazole, followed by manganese. Weaker sites are largely modeled with Br atoms. (*b*) Proteinase K crystals soaked with 4-iodopyrazole. Sites with the highest occupancies correspond to 4-iodopyrazole, while I atoms are modeled for many of the lower occupancy sites. Owing to the high quality of the data collected from the proteinase K crystals, several anomalous peaks correspond to S atoms. (*c*) RT–rilpivirine crystals soaked with 4-iodopyrazole. RT has a mixture of 4-iodopyrazole and I atoms throughout the occupancy range. Four S atoms were also detected.

The promiscuous binding of 4-bromopyrazole and 4-iodopyrazole allows the ordered binding of Br and I atoms, respectively, at many sites around a target protein. Both bromine and iodine scatter X-rays with strong anomalous signal when appropriate wavelengths are used (suitable choices include 0.92 Å for bromine and 1.54 Å for iodine). To determine whether the anomalous signal from the soaked compounds was sufficient for SAD phasing, data sets with a reflection-measurement multiplicity of greater than 7.0 were collected for crystals of HIV-1 RT, influenza endonuclease and proteinase K soaked with 100–500 m*M* compound for 10 min. Experimental phasing and model building were performed with *AutoSol* using the scaled data sets and the protein sequence. As shown in Table 1 ▸ and Fig. 6 ▸, the numbers of anomalous sites were 45 for RT–rilpivirine, 30 for proteinase K and 25 for endonuclease. For proteinase K, 11 sites were found to be sulfurs based on the presence of cysteine, methionine and sulfate at these positions in the refined structure (Fig. 6 ▸). High multiplicity was not necessary for successful SAD phasing, as a multiplicity of 3.2 (90° collected) was sufficient to solve the structure of proteinase K with *AutoSol* (Table 1 ▸), similar to that previously reported for 5-amino-2,4,6-triiodoisophthalic acid (Beck *et al.*, 2008 ▸).

**Figure 7:**
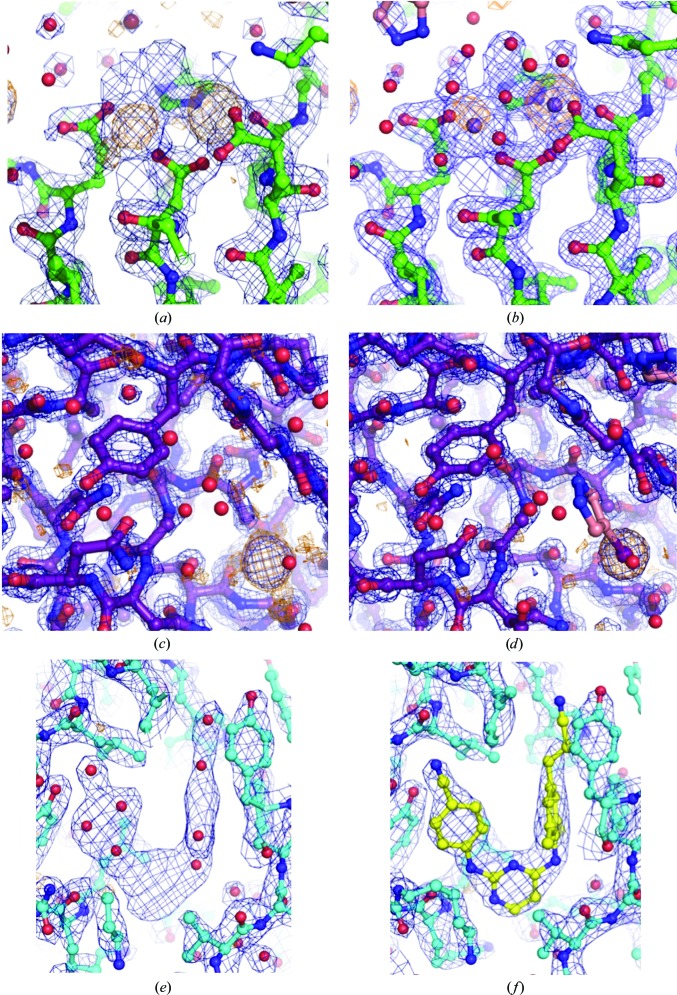
(*a*, *b*) Model and electron-density map for influenza endonuclease from *AutoSol* (*a*) and after refinement (*b*) with density (blue mesh; 2*F*_o_ − *F*_c_ coefficients) and anomalous difference contours [orange mesh; *F*(*hkl*) − *F*(−*h*−*k*−*l*) coefficients] shown at 2σ and 5σ, respectively. (*c*, *d*) Model and electron-density map for proteinase K from *AutoSol* (*c*) and after refinement (*d*) with 2*F*_o_ − *F*_c_ (blue mesh) and anomalous signal (orange mesh) contoured at 1.5σ and 6σ, respectively. (*e*, *f*) Model and electron-density map for HIV-1 RT–rilpivirine from *AutoSol* (*e*) and after refinement (*f*) with 2*F*_o_ − *F*_c_ (blue mesh) and anomalous difference contours (orange mesh) shown at 1.5σ and 5σ, respectively.

Further refinement of influenza endonuclease and proteinase K showed only modest improvement in electron-density map quality (Table 1 ▸ and Figs. 7 ▸ *a*–7 ▸ *d*) as only ligand/cofactor identification along with several rounds of reciprocal and real-space refinement were necessary. The lower resolution of the RT–rilpivirine data set (2.15 Å) as well as the complexity of the protein required additional rounds of refinement. However, the initial maps showed very clear electron density for the bound NNRTI, rilpivirine and the majority of the protein residues (Figs. 7 ▸ *e*, 7 ▸ *f* and Table 1 ▸).

### 3.5. Prediction of small-molecule fragment-screening success from 4-halopyrazole soaks   

**Figure 8:**
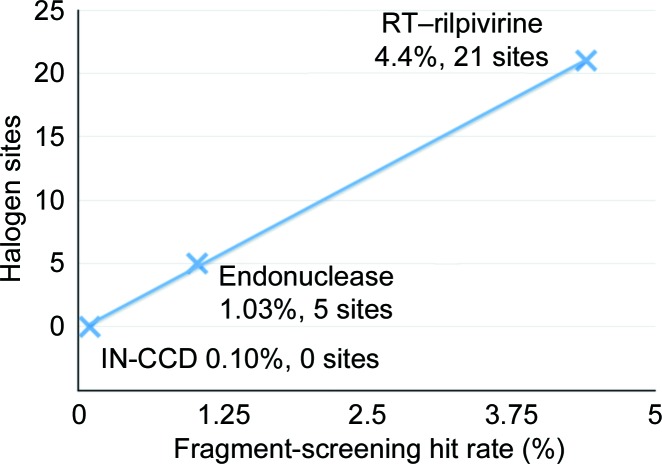
Comparison of the hit rate from X-ray crystallographic fragment screening and the number of strong, defined as clear electron density at 5σ in the refined anomalous difference maps, halogen-bound sites when crystals (RT–rilpivirine, influenza endonuclease and HIV-1 IN-CCD) are soaked with 4-bromopyrazole or 4-iodopyrazole at 500 m*M*.

Predicting the likelihood of success in soaking libraries of compounds into crystals has proven to be very difficult despite the considerable number of proteins screened. Hit rates from crystallographic screening can vary from ∼6 to 0% depending on the protein target, crystal form and crystallization conditions (Davies *et al.*, 2011 ▸). A third campaign of fragment screening by X-ray crystallography was recently completed in our laboratory against the catalytic core domain of HIV-1 integrase (Dyda *et al.*, 1994 ▸) (HIV-1 IN-CCD; to be published elsewhere) with an extremely low hit rate of 0.1%. Soaking of 500 m*M* 4-bromopyrazole into crystals of HIV-1 IN-CCD showed no binding to the protein, leading us to hypothesize that 4-bromo­pyrazole may also be a good predictor of success in crystallo­graphic fragment screening or for soaking specific small molecules into a protein crystal. Analysis of the number of halogen-bound sites *versus* the hit rate from crystallographic fragment screening indicates a strong correlation for the three targets discussed here (Fig. 8 ▸). However, further testing is needed to validate this intriguing hypothesis.

## 4. Conclusions   

4-Bromopyrazole was serendipitously found to be a promiscuous protein binder during a fragment-screening campaign. While in typical noncrystallographic compound screens such promiscuous binding would be ignored as not useful for drug discovery, in the case of crystallographic fragment screening it was possible to determine that the compound binds specifically at many sites throughout the proteins as opposed to binding through a nonspecific hydrophobic association/aggregation effect. 4-Bromopyrazole and 4-iodopyrazole have subsequently been shown to be useful tools for both ligand-binding hot-spot identification and as a source of anomalous signal to enable SAD phasing. The low cost ($55 per gram for 4-bromopyrazole and $18 per gram for 4-iodopyrazole at the time of preparation of this manuscript; Sigma–Aldrich), high solubility, availability and safety of the compounds make them convenient choices to include in a crystallographer’s toolbox.

## 5. Related literature   

The following references are cited in the Supporting Information for this article: Bauman *et al.* (2008 ▸).

## Supplementary Material

PDB reference: proteinase K, 5cw1

PDB reference: influenza endonuclease, 5cxr

PDB reference: HIV-1 reverse transcriptase, 5cym

PDB reference: 5cyq

Supplemental data and methods. DOI: 10.1107/S2052252515021259/jt5010sup1.pdf

## Figures and Tables

**Table 1:** Data-collection and refinement statistics Values in parentheses are for the highest resolution shell.

Protein	Proteinase K				Influenza endonuclease	HIV-1 RT	
Soak	500 m*M* 4-iodopyrazole				500 m*M* 4-bromopyrazole	20 m*M* 4-bromopyrazole	100 m*M* 4-iodopyrazole
PDB code	5cw1				5cxr	5cyq	5cym
Data collection							
Wavelength (Å)	1.5418				0.918	0.9177	1.5418
Unit-cell parameters (Å, °)	*a* = *b* = 68.02, *c* = 102.04, α = β = γ = 90				*a* = 87.90, *b* = 102.45, *c* = 66.06, α = β = γ = 90	*a* = 161.89, *b* = 73.17, *c* = 108.89, α = γ = 90, β = 100.56	*a* = 162.86, *b* = 73.40, *c* = 109.54, α = γ = 90, β = 100.70
Space group	*P*4_3_2_1_2				*C*222_1_	*C*2	
Data collected (°)	360	180	90	45	360	270	720
No. of frames	720	360	180	90	720	180	1440
Resolution range (Å)	35.0–1.45 (1.48–1.45)	50.0–1.45 (1.48–1.45)	50.0–1.45 (1.48–1.45)	35.0–1.50 (1.53–1.50)	50.0–2.00 (2.03–2.00)	50.0–2.15 (2.19–2.15)	50.0–2.10 (2.14–2.10)
Completeness (%)	99.5 (90.8)	99.2 (89.3)	98.2 (82.4)	85.0 (85.8)	100.0 (100.0)	98.1 (86.0)	98.6 (96.9)
Multiplicity	12.8 (6.9)	6.4 (3.5)	3.2 (1.9)	1.9 (1.7)	7.7 (7.6)	2.6 (1.9)	7.1 (6.9)
Anomalous completeness (%)	99.5	99.1	97.3	75.5	99.9	92.4	98.7
〈*I*/σ(*I*)〉	55.7 (4.8)	42.7 (3.5)	28.9 (2.5)	24.2 (4.2)	26.4 (2.7)	12.8 (1.1)	25.1 (2.1)
*R*_sym_	0.051 (0.426)	0.047 (0.368)	0.043 (0.371)	0.031 (0.200)	0.086 (0.854)	0.075 (0.625)	0.068 (0.937)
*R*_p.i.m._	0.014 (0.171)	0.019 (0.216)	0.027 (0.293)	0.025 (0.168)	0.033 (0.331)	0.054 (0.539)	0.027 (0.380)
*R*_meas_	0.053 (0.461)	0.050 (0.430)	0.051 (0.475)	0.040 (0.263)	0.093 (0.916)	0.093 (0.829)	0.072 (>1.0)
*AutoSol* results							
Sites	30	26	24	12	25		46
FOM	0.432	0.385	0.340	0.267	0.329		0.240
Overall score	49.46 ± 9.01	48.34 ± 9.30	43.20 ± 9.82	25.35 ± 14.42	39.87 ± 11.66		40.34 ± 11.25
*R*_work_	0.175	0.175	0.192	0.494	0.236		0.256
*R*_free_	0.203	0.1923	0.216	0.507	0.266		0.299
Waters	309	335	363	205	139		512
Residues/refined	278/279	278/279	264/279	96/279	185/187		810/902
Mean map CC	0.964	0.961	0.954	0.557	0.919		0.898
Refinement statistics							
Total No. of atoms	4437				3507	16521	16186
No. of solvent atoms	398				149	352	236
No. of reflections	42913				20422	131079	144235
Reflections in *R*_free_ set	2156				1044	3847	7334
Completeness (%)	99.45				99.78	97.7	98.4
Completeness (*R*_free_ set) (%)	5.02				5.11	2.93	5.08
*R*_work_	0.131				0.175	0.203	0.195
*R*_free_	0.154				0.198	0.224	0.229
R.m.s.d., bond lengths (Å)	0.005				0.006	0.003	0.007
R.m.s.d., bond angles (°)	0.999				0.810	1.15	1.02
Ramachandran statistics							
Residues in favored regions	97.2				96.0	96.6	97.45
Residues in disallowed regions	0.0				0.0	0.0	0.11
