# Supplementary material for: Rapid experimental SAD phasing and hot-spot identification with halogenated fragments
Source: IUCrJ. 2016 Jan 1;3(Pt 1):51–60. doi: 10.1107/S2052252515021259 (PMC4704079; doi:10.1107/S2052252515021259)
Supplement: Supplementary file 1 [file m-03-00051-sup1.pdf]

IUCrJ

Volume 3 (2016)

Supporting information for article:

Rapid experimental SAD phasing and hot-spot identification with halogenated fragments

Joseph D. Bauman, Jerry Joe E. K. Harrison and Eddy Arnold

S1. Methods

S1.1. Expression, purification, and crystallization of HIV-1 RT

HIV-1 RT construct RT52A (crystallization optimized mutant) was expressed/purified/crystallized as described previously (Bauman et al., 2008; Bauman et al., 2013). A plasmid encoding RT52A was transformed into BL21-CodonPlus®-RIL (Stratagene, La Jolla, CA) competent cells and grown on LB-agar plates containing 35 mg/liter streptomycin and 0.1% (w/v) glucose. A single colony was picked and grown overnight in LB + 35 mg/liter streptomycin and 0.5% glucose at 37°C with shaking. The overnight culture was diluted 100-fold into a culture of 4 liters and incubated at 37°C with shaking. At an OD_{600} of 0.9, the cells were induced with 1 mM IPTG and incubated for three hours prior to pelleting and storage at -80°C.

Nickel column purification was performed according to the manufacturer's recommendations (Qiagen, Germantown, MD) with the following modifications: no lysozyme was added to the lysate, 600 mM NaCl instead of 300 mM was used in each of the standard buffers, 0.1% (v/v) Triton X-100 was added to the lysate and wash buffers, and an extra high-salt wash step was performed with 1.2 M NaCl. Following elution, the yield of RT was determined by OD_{280} (OD_{280}/3.1 x dilution factor) and a 1:100 (weight ratio) of HRV14 3C protease to RT was added. The protease-containing reaction was incubated at 4°C overnight. The solution was buffer exchanged 20-fold into buffer A (50 mM diethanolamine pH 8.9) using an Amicon Ultra-15 Centrifugal Filter unit with an Ultracell-30 membrane (Millipore). The solution was filtered with a 0.22 micron filter, and 10-20 mg was loaded onto a monoQ 10/100 column (Amersham Biosciences, Piscataway, NJ) equilibrated with buffer A. The column was washed with buffer A and the samples eluted over one hour by running a gradient from 0 to 25% buffer B (buffer A + 1 M NaCl) at a flow

rate of 4 ml/minute. The RT was buffer exchanged and concentrated to 20 mg/ml in 10 mM Tris pH 8.0 and 75 mM NaCl. The concentrated RT was used immediately for crystallization or aliquoted and stored at -80°C.

RT crystallization was performed using the sitting drop method with 96-well crystallization trays (Qiagen, Valencia, CA). Prior to crystallization, RT52A (20 mg/ml) was incubated with rilpivirine (TMC278/Edurant) at 1:1.5 protein to drug molar ratio at room temperature (~23°C) for 30 minutes. RT52A-rilpivirine crystals were produced in hanging drops at 4°C with a 1:1 ratio of protein and well solution containing 11% (v/v) PEG 8000, 4% (v/v) PEG 400, 50 mM imidazole pH 6.6, 10 mM spermine, 15 mM $MgSO_4$, 100 mM ammonium sulfate, and 5 mM tris(2-carboxyethyl)phosphine together with an experimentally optimized concentration of microseeds from previously generated and crushed RT52A-rilpivirine crystals (preseeding).

S1.2. Expression, purification, and crystallization of influenza endonuclease

The influenza endonuclease construct (Bauman et al., 2013b) was expressed in BL21 (RIL) cells (Stratagene, La Jolla, CA). The BL21 cells were grown to an OD_{600} of 0.8 in 2xYZ media and induced with 0.15 mM IPTG at 17°C for 17 hours. Cells were harvested by centrifugation and purified on a Ni-NTA (Qiagen, Valencia, CA) column according to manufacturer's recommendations except the final elution was performed with 500 mM imidazole present. The tandem-hexahistidine tag was then removed by HRV14 3C protease cleavage followed by an additional IMAC purification step. Endonuclease was further purified by size-exclusion chromatography using HiLoad 26/60 Superdex 75 (GE Healthcare, Piscataway, NJ). The protein was concentrated to 10 mg/ml using an Ultrafree 10K filter (Millipore), aliquoted and stored in 100 mM NaCl and 20 mM Tris pH 8.0 at -80°C.

Endonuclease was crystallized using the sitting drop method with 96-well crystallization trays (Qiagen, Valencia, CA). 5 mg/ml endonuclease was mixed in equal volumes of precipitant solution containing 1 mM manganese chloride, 200 mM MES pH 6.7, 25% (v/v) PEG 8000, 100 mM ammonium sulfate, 10 mM magnesium acetate, 10 mM taurine, and 50 mM sodium fluoride. All crystallization was performed at 20°C.

S1.3. Crystallization of proteinase K

Proteinase K was purchased from Sigma-Aldrich (St. Louis, MO) and crystallized as described previously (Beck et al., 2010). 20 mg/ml proteinase K was mixed with an equal volume of 100 mM Tris pH 7.2 and 1.28 M ammonium sulfate.

S1.4. Ligand soaks

Compounds were purchased from Sigma-Aldrich (St. Louis, MO) or Acros (Geel, Belgium).

The RT compound/cryo soaking solutions were prepared with crystallization well solution with the addition of 80 mM L-arginine, 5% (v/v) ethylene glycol, and 20% (v/v) DMSO (containing compound). 80 mM L-arginine was included to improve the solubility of the ligands (Bauman et al., 2013). Crystals of RT52A-rilpivirine were harvested three months after crystals formed. The crystals were placed in compound/cryo soaking drops for ten minutes before flash freezing in liquid nitrogen.

Influenza endonuclease ligand soaks were performed by gradient shifting, over 20 minutes, the crystal solution to 1 mM manganese sulfate, 200 mM HEPES pH 7.7, 25% (w/v) PEG 8000, 50 mM ammonium sulfate, 5 mM magnesium acetate, 10% (v/v) DMSO, and 5% (v/v) ethylene glycol. Compounds, in DMSO, were soaked into crystals (final

volume of 10% and concentration of 10 mM) for 2 hours before flash freezing in liquid nitrogen.

Proteinase K ligand soaks were performed by addition of 500 mM iodopyrazole directly to well solution with the addition of 30% (v/v) glycerol for cryoprotection. Crystals were soaked in the ligand solution containing cryoprotectant for ten minutes before flash freezing in liquid nitrogen.

S1.5. Data collection and processing

X-ray diffraction data collection was performed at the Cornell High Energy Synchrotron Source (CHESS) F1 beamline, the National Synchrotron Light Source (NSLS) beamline X25, and the CABM Macromolecular X-ray Crystallography Facility. The diffraction data were indexed, processed, scaled, and merged using *HKL2000* (Otwinowski et al., 1997). Experimental phasing, structure refinement, and model building were carried out using PHENIX (1.8.2-1988) (Adams et al., 2010) and Coot (0.8.1) (Emsley et al., 2010).

S1.6. DFT calculations

The geometry was fully optimized for each compound in its singlet ground state using M06 functional (Zhao et al., 2008) as implemented in Spartan'14 v1.1.0 (Spartan'14, Wavefunction Inc, Irvine, CA) with the 6-311+G** basis set, which in the valence space is of triple-zeta quality and of double-zeta-quality polarization functions (Glukhovstev et al., 1995; Yao et al., 2007; Ribieto et al., 2012; Shallangwa et al.,2014). The structures were verified to be at a minimum energy without any imaginary frequencies. Single point energy calculations were computed using the 6-311++G** basis set with a diffuse function on all the atoms including the hydrogens using the optimized structures. Electrostatic potential

energy maps were generated at a 0.002 (arbitrary unit) isosurface. The electrostatic surface potential (ESP) provides a charge density distribution which gives a visual indication of probable interactions of a point-like charged species with organic molecules (Naray-Szabo et al., 1995; Mircescu et al., 2011).

**Compound name**	**Electrostatics**	**Number of sites on RT**
4-iodopyrazole	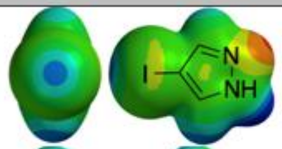	21
4-bromopyrazole	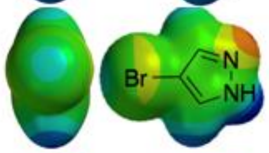	15
2-bromopyrimidine	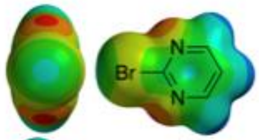	4
iodopyrazine	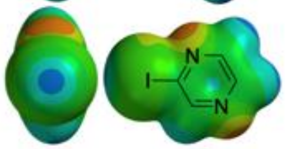	2
3-iodopyrrole	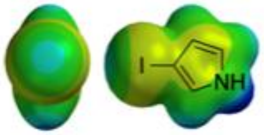	0
3-bromopyrrole	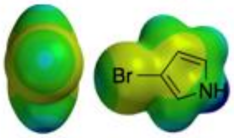	0
4-iodopyridine	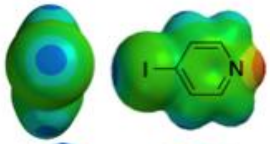	0
4-bromopyridine	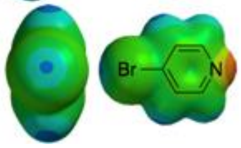	0

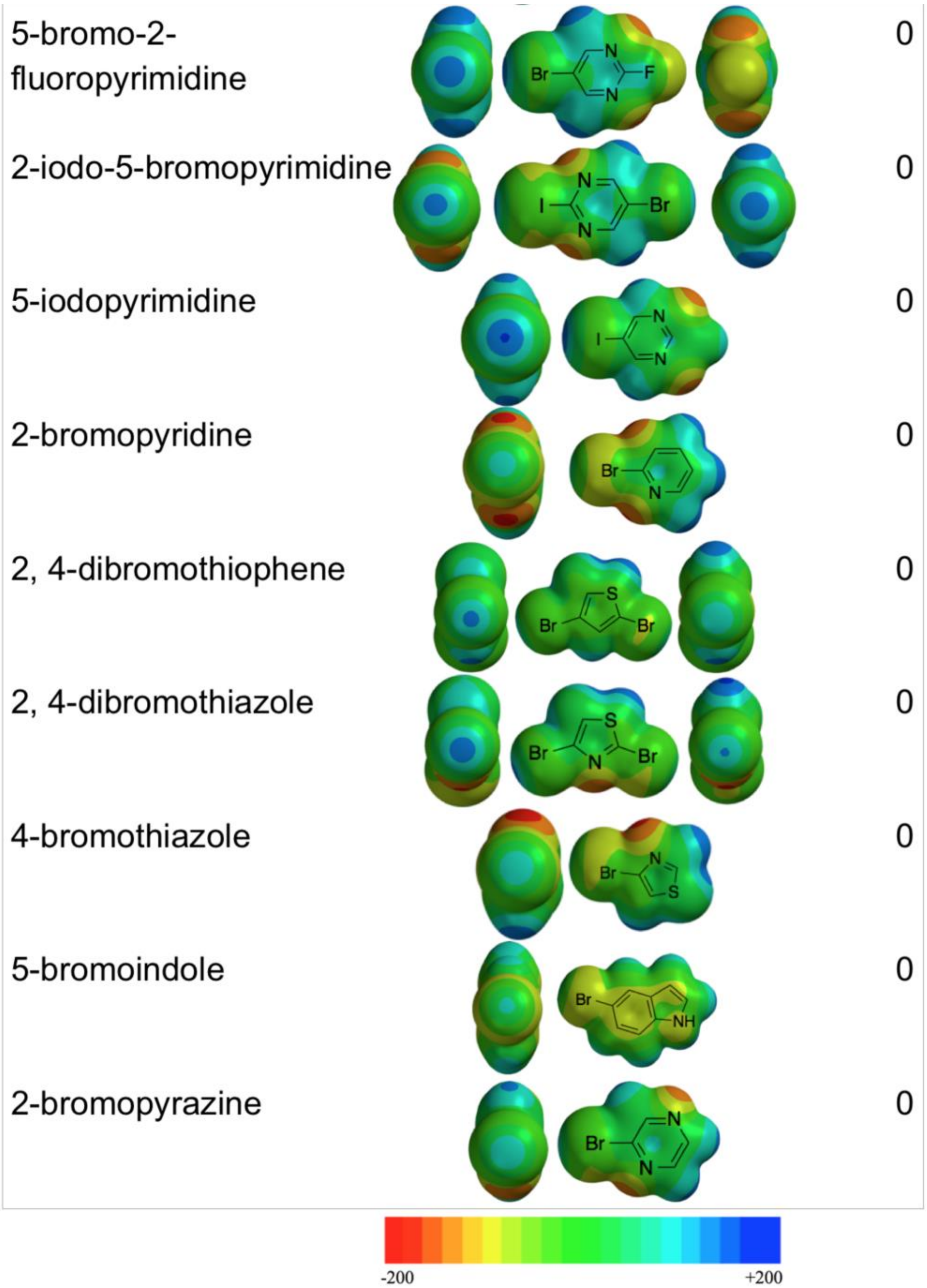

Figure S1 Calculated electrostatic potential surfaces for 4-bromopyrazole, 4-iodopyrazole, and related analogs. Full table of derivatives tested for binding to RT-rilpivirine with density functional theory calculations of the electrostatic potential energy surface.

References

Adams, P. D., Afonine, P. V., Bunkóczi, G., Chen, V. B., Davis, I. W., Echols, N., Headd, J. J.,

Hung, L.-W., Kapral, G. J., Grosse-Kunstleve, R. W., McCoy, A. J., Moriarty, N. W., Oeffner, R., Read, R. J., Richardson, D. C., Richardson, J. S., Terwilliger, T. C. & Zwart, P. H. (2010). *Acta Crystallogr D Biol Crystallogr*. **66**, 213–221.

Bauman, J. D., Das, K., Ho, W. C., Baweja, M., Himmel, D. M., Clark, A. D., Jr., Oren, D. A., Boyer, P. L., Hughes, S. H., Shatkin, A. J., & Arnold E. (2008). *Nucleic Acids Res*. **36**, 5083–5092.

Bauman, J. D., Patel, D., Dharia, C., Fromer, M., Ahmed, S., Frenkel, Y., Eck, J. T., Ho, W., Das, K., Shatkin, A. J. & Arnold E. (2013a). *J Med Chem.* **56**, 2738-2746.

Bauman, J. D., Patel, D., Baker, S. F., Vijayan, R. S. K., Xiang, A., Parhi, A. K., Martínez-Sobrido, L., Lavoie, E. J., Das, K. & Arnold, E. (2013a). *ACS Chem Biol*. **8**, 2501-2508.

Beck, T., Gruene, T. & Sheldrick, G. M. (2010). *Acta Crystallogr D Biol Crystallogr*. **66**, 374–380.

Emsley, P., Lohkamp, B., Scott, W. G. & Cowtan, K. (2010). *Acta Crystallogr D Biol Crystallogr*. **66**, 486–501.

Glukhovtsev, M. N., Pross, A., McGrath, M. P. & Radom, L. (1995). *J Chem Phys*. **103**, 1878–1885.

Mircescu, N. E., Varvescu, A. & Herman, K. (2011). *Spectrosc.* **26**, 311-315

Naray-Szabo, G. & Ferenczy, G. G. (1995). *Chem Rev*. **95**, 829–847.

Otwinowski, Z. & Minor, W. (1997). *Method Enzymol*. **276**, 307–326.

Ribeiro, R. D. S., Esteves, P. M. & Mattos, M. C. S. de (2012). *J Braz Chem Soc*. **23**, 228–235.

Shallangwa, G. A., Uzairu, A., Ajibola, V. O. & Abba, H. (2014). *ISRN Phys Chem*. **2014**, 1–8.

Yao, L., Du, L., Ge, M., Ma, C. & Wang, D. (2007). *J Phys Chem A.* **111**, 10105-10110.

Zhao, Y. & Truhlar, D. G. (2008). *Theor Chem Account* **120**, 215–241.
